# Supplementary material for: The lexicon of antimicrobial peptides: a complete set of arginine and tryptophan sequences
Source: Commun Biol. 2021 May 21;4:605. doi: 10.1038/s42003-021-02137-7 (PMC8140080; doi:10.1038/s42003-021-02137-7)
Supplement: Supplementary file 2 — Supplementary Information [file 42003_2021_2137_MOESM2_ESM.pdf]

Supplementary Information for

The Lexicon of Antimicrobial Peptides: a Complete Set of Arginine and Tryptophan Sequences.

Authors: Sam Clark, Thomas A Jowitt, Lynda K Harris, Christopher G Knight and Curtis B Dobson.

Supplementary Figures 1-5

Supplementary Tables 1 and 2

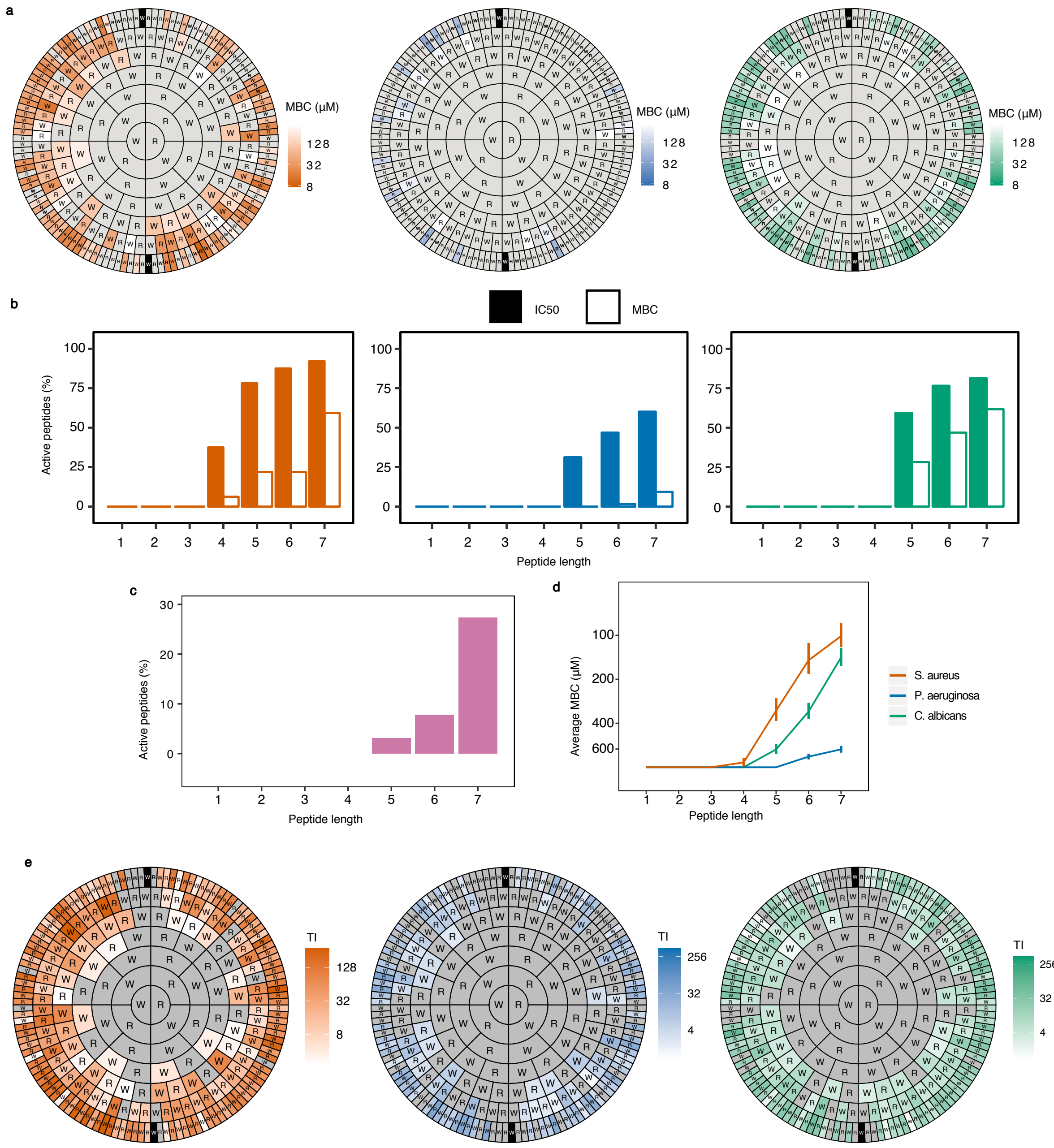

Supplementary Fig. 1: Microbicidal activity of the complete set of peptides comprised of W and R up to 7 residues long. (a) Microbiocidal activities against the 3 different microorganisms studied (*S. aureus*, *P. aeruginosa* and *C. albicans*), represented using Harris-Clark diagrams. Grey sections represent peptides which did not exhibit an MBC within the range of concentrations assayed (0.8 – 400 µM). (b) Comparison of the percentage of peptides in each length subset which exhibited inhibitory or microbiocidal activity against the 3 different microorganisms within the concentration range assayed. (c) The percentage of peptides in each length subset that exhibited hemolytic activity within the concentration range assayed. (d)

Effect of peptide length on harmonic means and standard deviations of MBC. For further explanation see Fig. 1. All error bars shown are +/- s.d. (n = 2, 4, 8, 16, 32, 64 and 128 peptides for lengths 1 – 7 respectively). (e) Therapeutic indices against the three microorganisms represented using Harris-Clark diagrams. Grey sections represent peptides which exhibited neither an IC_{50} nor an EC_{50} within the range of concentrations assayed (0.8 – 400 µM).

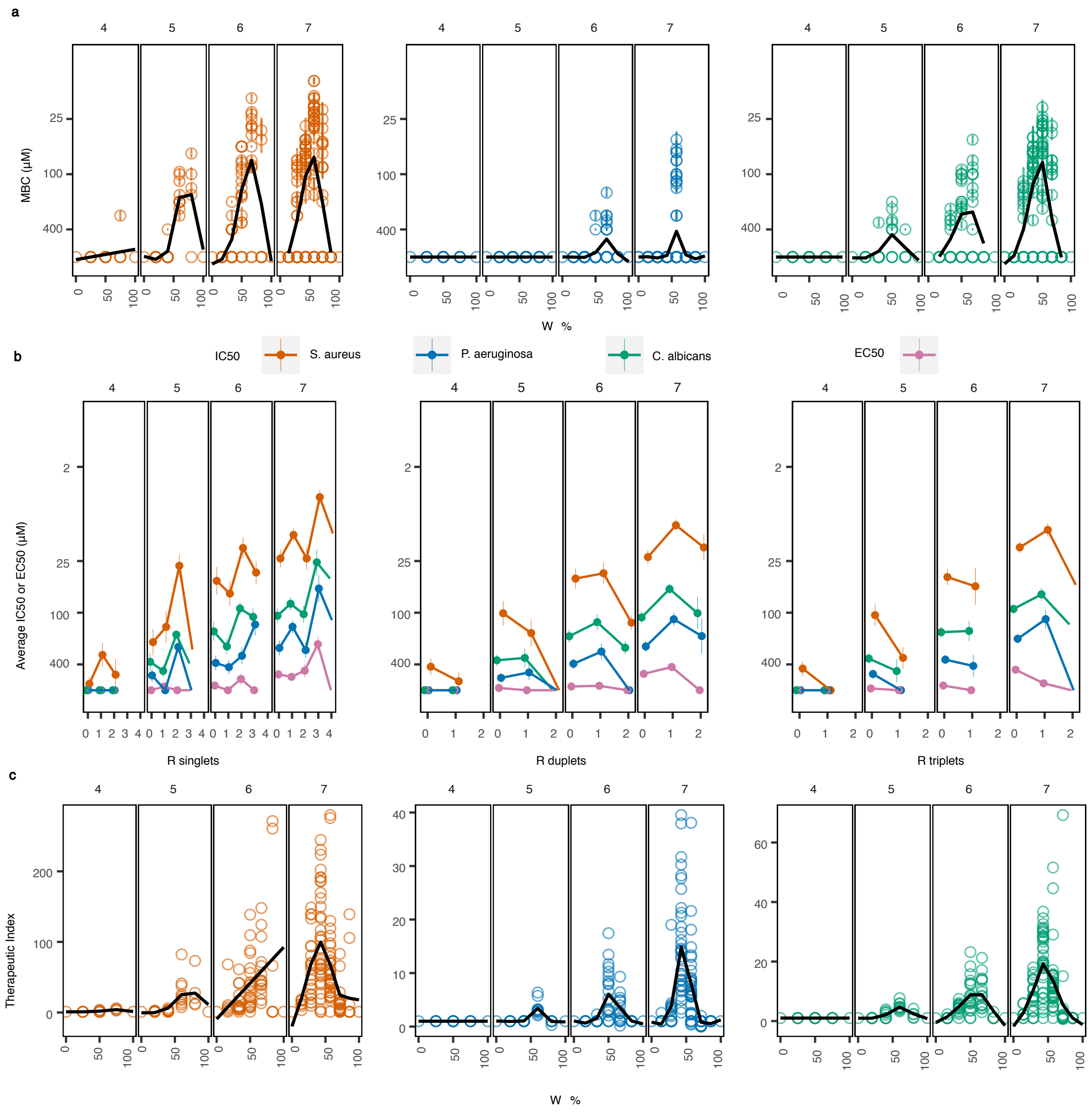

Supplementary Fig. 2: Effect of various peptide primary structural features on microbicidal activity. (a) Microbiocidal activity plotted against percentage W residues within the sequence, faceted by peptide length (indicated at the top of each sub panel). The black spline through the data indicates the average activity for each peptide length. Error bars shown are +/- s.e.m. (b) Analysis of average inhibitory and hemolytic activities for peptides with

different numbers of isolated R singlets (R), duplets (RR), and triplets (RRR), faceted by peptide length (indicated at the top of each sub panel). Error bars shown are +/- s.e.m. For further explanation see Fig. 2. (c) Therapeutic index plotted against percentage W residues within the sequence, faceted by peptide length (indicated at the top of each sub panel). The black spline through the data indicates the average therapeutic index for each peptide length. Error bars shown are +/- s.e.m. (n=4).

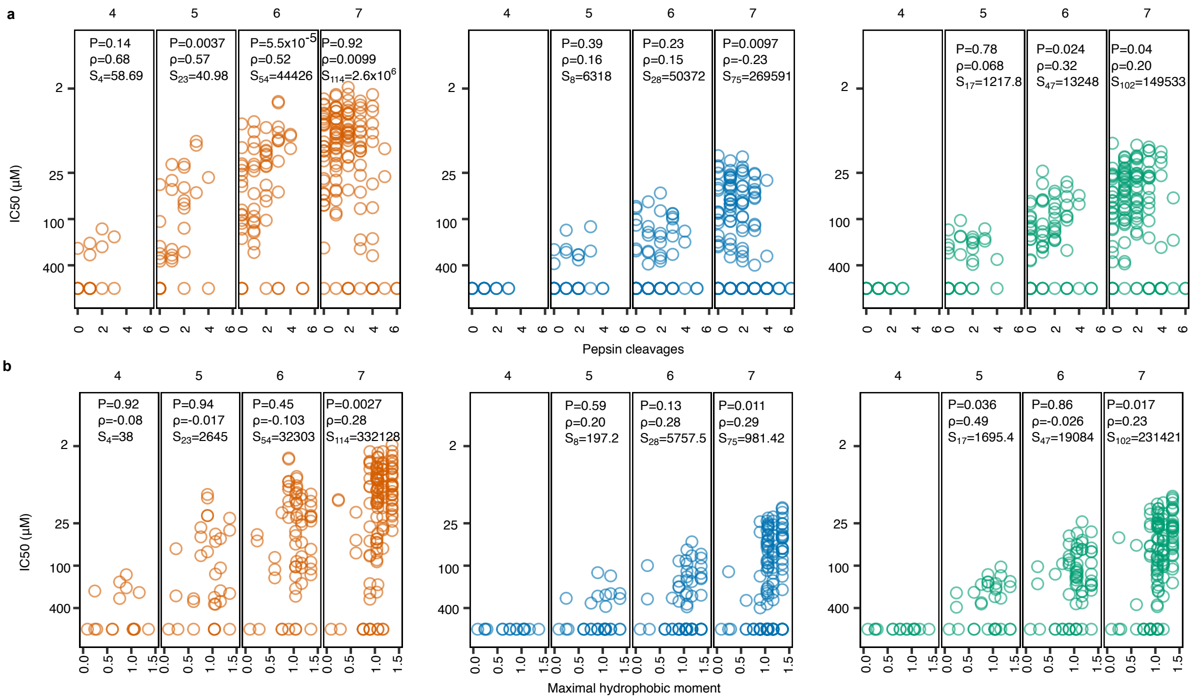

Supplementary Fig. 3: *In silico* analysis of effect of number of pepsin cleavage sites or hydrophobic moment on antimicrobial activity. (a) Inhibitory activity plotted against number of potential pepsin cleavage sites or against (b) hydrophobic moment, faceted by peptide length. Spearman's rank correlation coefficients were calculated for each faceted dataset for peptides which exhibited IC_{50} within the concentration range assayed (0.8 – 400 μM) and are shown in each panel.

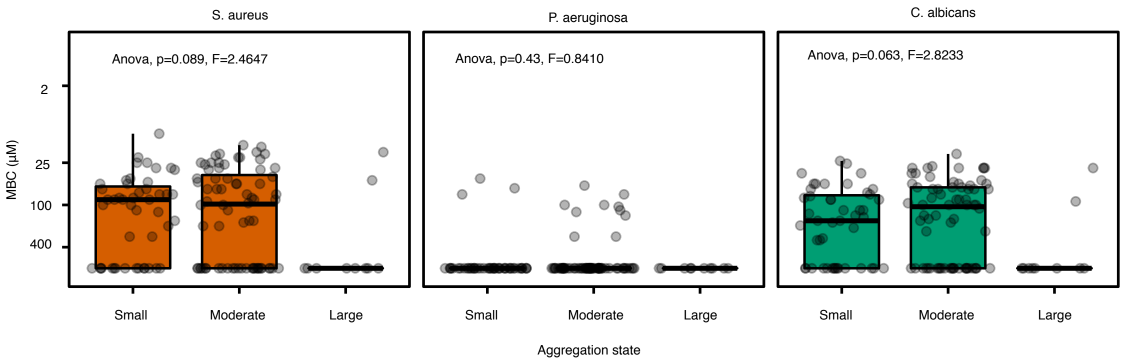

Supplementary Fig. 4: Relationship between microbiocidal activity and peptide aggregation. (a) Comparisons of microbiocidal activity and aggregation state for the three organisms assayed. Aggregation state was assessed in stock solutions (800 µM) using DLS, indicating the three size categories identified (featuring small, moderate or large aggregates). Error bars shown are +/- s.e.m. For further explanation see Fig. 3.

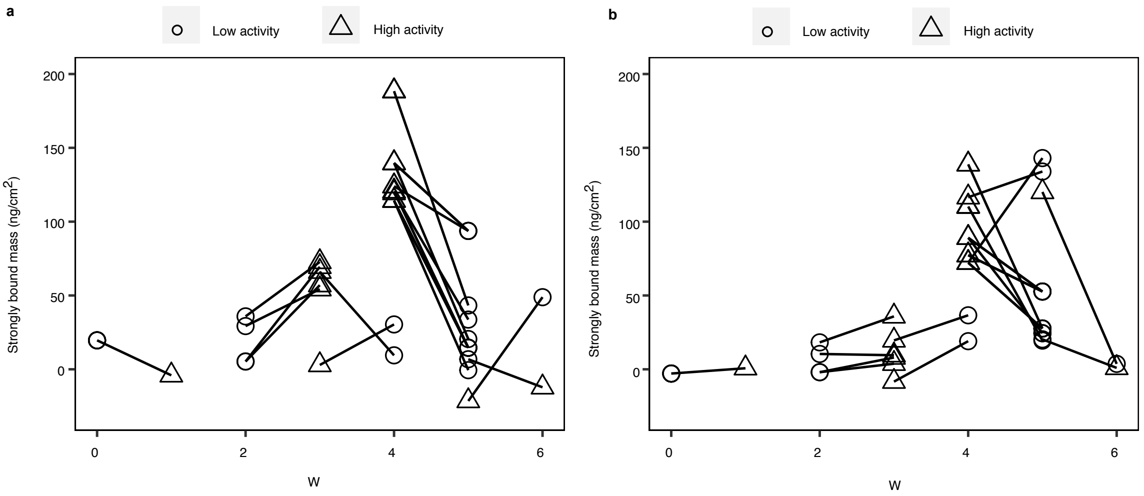

Supplementary Fig. 5: Relationships between selected peptide pairs and membrane binding. Mass of peptides bound to (a) anionic and (b) neutral membranes relative to the total number of W residues in each peptide. Peptide pairs in the selection are connected by lines, with the less active antimicrobial peptide indicated by a circle and the more active antimicrobial peptide by a triangle. For further explanation see Fig. 4.

Supplementary Table 1: features selected by the Boruta algorithm as having a statistically significant relationship with each of the response indices. For each index, the features have been ordered in descending level of mean importance. The number of features selected is shown, as well as the number of iterations required to reach a decision for all features.

	**IC_{50}**			**MBC**			**EC_{50}**	**OD_{vis}**
	S. aureus	P. aeruginosa	C. albicans	S. aureus	P. aeruginosa	C. albicans		
	mass	R%	mass	mass	mass	mass	mass	W
	$\bar{X}_R$	W%	R%	X_7	sequence	W	W	mass
	length	W	W%	length	W	W%	WW	R%
	W%	mass	W	W	$\bar{X}_W$	R%	R%	W%
	R%	R	length	R%	R%	σ_W	W%	length
	W	length	X_5	W%	W%	R	R	R
	σ_W	sequence	σ_W	σ_W	R	length	WWRRWW	
	X_5	σ_R	R	σ_R	RRRW	σ_R	RWW	
	X_6	$\bar{X}_R$	σ_R	WW	WWRRRW	sequence	σ_R	
	sequence	σ_W	X_6	R	RRR	WW	length	
	RWWWWW	X_7	WW	WWR	$\bar{X}_R$	X_6	X_7	
	$\bar{X}_W$	X_5	$\bar{X}_R$	$\bar{X}_R$	RWRR	WWRRW	RWWR	
	WWWWW	X_6	sequence	RRWW	X_6	RWW	X_6	
	R	$\bar{X}_W$	WWR	RWRWWW		WWR	σ_W	
	X_4	RRWW	X_7	RRWWRR		$\bar{X}_R$	WWR	
	WR	WWR	WRWWWW	RW		$\bar{X}_W$	RRW	
	σ_R	RWW	$\bar{X}_W$	RWW		X_7	WWRWW	
	WWWWWR	WW	RWWWWW	$\bar{X}_W$		WWW	sequence	
	RW	RW	RWW	X_5		WWRRR	RWWRWW	
	X_7	RRW	WR	WWRW		RWWW	WWRRW	
	WRWWWW	RWR	WWWWW	sequence		X_5	WWWWR	
	WWRW		WWRR			RW		
	WWR		WRR			WRR		
	WWW		RWWWW			WWRR		
	WW		X_4			WRRW		
	WWRWW		RRWW			RRWW		
	WRRR					RRW		
	RWW					WRWWR		
	WRR					RWWWW		
						WWRRRR		
**features selected**	29	21	26	21	13	30	21	6

Supplementary Table 2: Table showing the feature space used to describe peptides. Features have been split into different categories. The numbers of features in each category are shown, along with the total number of features in the space. In the symbol column A is amino acid and C is amino acid class.

**Symbol**	**Feature category**	**Number of features**
A%	Percentage abundance of amino acid	2
$\bar{X}_A$	Mean position of amino acid	2
σ_A	Positional standard deviation of amino acid	2
AA	Duplets	4
AAA	Triplets	8
X_7	Sequence position	7
	Longer sub-sequences	240
	Other features (length, mass)	2
	**Total number of features**	**267**
