# Supplementary material for: The lexicon of antimicrobial peptides: a complete set of arginine and tryptophan sequences
Source: Commun Biol. 2021 May 21;4:605. doi: 10.1038/s42003-021-02137-7 (PMC8140080; doi:10.1038/s42003-021-02137-7)
Supplement: Supplementary file 3 — Description of Additional Supplementary Files [file 42003_2021_2137_MOESM3_ESM.pdf]

Description of Additional Supplementary Files

File Name: Supplementary Movie 1
Description: **Antimicrobial activity network**. Peptides are nodes in the network and peptides separated by a single edit (single residue insertion, deletion or base change) are connected by an edge. Nodes are represented by red spheres, whose radius is proportional to the antimicrobial activity against *S. aureus* ($1/IC_{50}$).
